# Multicolor upconversion luminescence of dye-coordinated Er^3+^ at the interface of Er_2_O_3_ and CaF_2_ nanoparticles

**DOI:** 10.1080/14686996.2018.1558911

**Published:** 2018-12-18

**Authors:** Ayumi Ishii, Yuya Adachi, Ayaka Hasegawa, Miyu Komaba, Shuhei Ogata, Miki Hasegawa

**Affiliations:** a College of Science and Engineering, Aoyama Gakuin University, Sagamihara, Kanagawa, Japan; b JST, PRESTO, Kawaguchi, Japan

**Keywords:** Upconversion, nanoparticle, interfacial complex, lanthanide, squaraine dye, 40 Optical, magnetic and electronic device materials, 102 Porous / Nanoporous / Nanostructured materials, 204 Optics / Optical applications, 505 Optical / Molecular spectroscopy, 212 Surface and interfaces

## Abstract

Multicolor upconversion luminescence of Er^3+^ was successfully enhanced by optimizing the interface in dye-coordinated nanoparticles with a core/shell structure. Red and green upconversion emissions of Er^3+^ were obtained at the interface of oxide nanoparticles via the intramolecular energy transfer from the coordinating squaraine dye with high light-absorption ability, which was more efficient than emissions through the energy transfer from metal ions such as Yb^3+^. Additionally, CaF_2_ nanoparticles as a core material minimized the energy loss with nonradiative downward relaxations in Er^3+^, resulting in the observation of unusual blue upconversion emissions from the upper energy level of Er^3+^ by nonlaser excitation using a continuous-wave (CW) Xe lamp at an excitation power of 1.2 mW/cm^2^.

## Graphical Abstract

**Figure:**
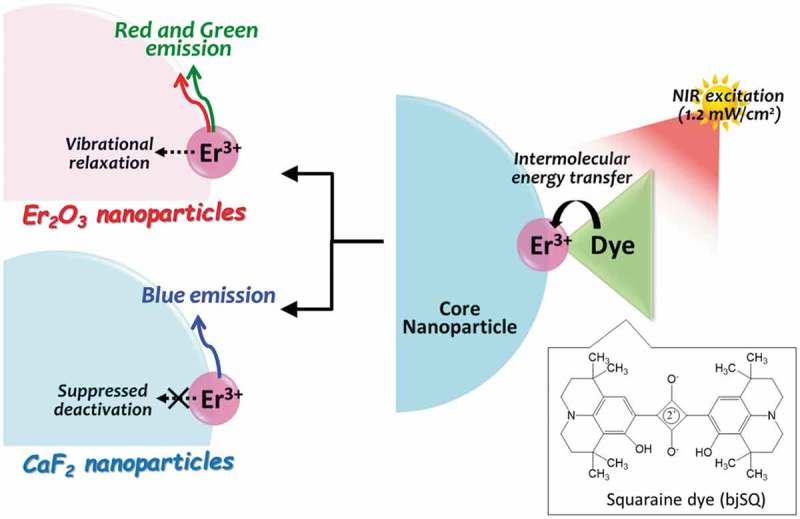

## 1. Introduction

The upconversion phenomenon, which can convert two or more low-energy photons into one high-energy photon, has great potential for effective light energy use. Lanthanide (Ln)-based upconversion materials have attracted great attention in applications such as displays, biosensing, lighting, and solar cells [1–4]. In general, the upconversion emission of Ln ions occurs through the metal-to-metal multiple energy transfer from near-infrared (IR) light absorbers (Yb^3+^) to visible light emitters (Tm^3+^, Er^3+^, Ho^3+^, Nd^3+^, etc.), in which the emission color strongly depends on the dopant concentration [5], dispersing solvent [6], core/shell structure [7], and host material [8]. For instance, Er^3+^ has some energy levels from the blue to near-IR spectral region, and it is well-known that green and red emissions are most commonly observed under 980 nm excitation of Yb^3+^ as a co-dopant [9], while blue emission rarely occurs through a three-photon process [10]. Most investigations have reported the control of the green/red emission ratio by varying the doping concentration of Yb^3+^ and Er^3+^ in a dielectric host lattice such as NaYF_4_ [11–15]. In such metal-to-metal energy transfer-based upconversion systems, the distance between dopants and the light-absorption ability are the keys to enhancing the upconversion emission. However, Ln-doped upconversion nanoparticles have some limitations to obtaining the desired emission colors, especially blue emission, since their forbidden transitions of 4f-4f configurations have a low light-absorption coefficient (*ɛ *= 1–100 dm^3^mol^−1^cm^−1^) [16] and nonradiative downward relaxations often take place with the upward transitions.

**Figure 1.**
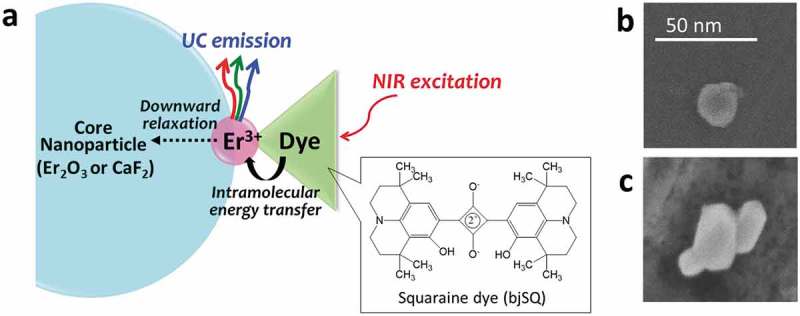
(a) Schematic of the enhancement of upconversion (UC) emission by dye-coordinated Ln nanoparticles. SEM images of bjSQ-coordinated (b) Er oxide nanoparticles and (c) CaF_2_/Er nanoparticles.

In this investigation, an active interface composed of dye-coordinated Ln nanoparticles with a core/shell structure has been developed to control multicolor upconversion luminescence of Er^3+^ (Figure 1(a)), where the emissions of Er^3+^ can be enhanced mainly by two efficient energy migration pathways at the interface: (1) the intramolecular energy transfer from coordinating dyes and (2) the nonradiative downward relaxation on core nanoparticles. Unlike Ln^3+^ with its low light-absorption coefficient, organic dyes have a high ability to absorb light, and the excitation energy of the dye can be transferred efficiently to the Ln ion by the formation of coordination bonds. Previously, we successfully developed a novel upconversion emission system composed of core/shell structured Tm/Yb oxide nanoparticles coordinated with indigo dyes [17]. This system achieved upconversion blue emissions by excitation with a continuous-wave (CW) Xe lamp at an excitation power of less than solar irradiation power at 640 ± 5 nm (1.4 mW/cm^2^), which was induced through the intramolecular energy transfer pathway from the organic dyes to emissive Ln ions.

Here, the squaraine dye, 2,4-bis[8-hydroxyl-1,1,7,7-tetramethyl-julolidin-9-yl]squaraine (bjSQ), having a metal coordination site, was selected as a photosensitizer to enhance the upconversion emission of Er^3+^ (Figure 1(a)). Squaraine dyes, with their unique aromatic four-membered ring system derived from squaric acid, are a class of organic dyes that exhibit intense absorption (absorption coefficient: *ɛ* ≈ 3 × 10^5^ cm^−1^M^−1^) and fluorescence bands (luminescence quantum yield: *ϕ *≈ 0.65), typically in the red and near-IR regions [18]. Two core nanoparticles with different vibration modes (Er_2_O_3_ and CaF_2_) were selected to construct the interface of the dye-coordinated nanoparticles. In the oxide state, which is the most stable in Ln compounds, the surface conditions may strongly affect the relaxation pathways of Ln^3+^ since the vibration energy of the hydroxyl group (-OH) in adsorbed (or hydrated) H_2_O on the surface matches well with some transition energies of Ln^3+^. On the other hand, CaF_2_ nanoparticles as the core can suppress the vibration deactivation pathway in the upconversion process due to their low phonon energy, high refractive index, optical transparency, and high chemical and thermal stabilities [19–22].

## 2. Experimental section

### 2.1. Sample preparation

Er_2_O_3_ nanoparticles with a diameter of approximately 20 nm (Kanto Chemical Co., Inc., Japan) were sintered at 200 °C for 20 min in the presence of nitrogen to remove the water absorbed at the surface. The squaraine dye (2,4-bis[8-hydroxyl-1,1,7,7-tetramethyljulolidin-9-yl]squaraine, Tokyo Chemical Industry Co., Ltd., Japan) was dissolved into chloroform solution (2.5 mM) and reacted with Er_2_O_3_ nanoparticles (1 wt%). The colloidal suspension was stirred for 60 min at 25 °C. After filtration, the resulting bright green nanoparticles were rinsed with hexane and dried under vacuum. Er/Yb oxide nanoparticles were prepared as the previous method: The Er_2_O_3_ nanoparticles (10 wt%) were immersed in a 10 mM ethanol solution of YbCl_3_.6H_2_O (Kanto Chemical Co., Inc.) and stirred at 70 °C for 60 min. After filtration, the nanoparticles were baked from 110 to 400 °C at a rate of about 15 °C and kept for 60 min. The chloride ions on the surface of nanoparticles were almost excluded by the calcination process over 400 °C [17]. CaF_2_ nanoparticles were synthesized by calcium nitrate (Ca(NO_3_)_2_) and ammonium fluoride (NH_4_F). Ca(NO_3_)_2_.4H_2_O (Wako Pure Chemical Industries Ltd., Japan) was dissolved in water (75 mM) with ethylenediaminetetraacetic acid (EDTA, 7.5 mM) and refluxed for 60 min. The resulting solution was reacted with NH_4_F (150 mM) and refluxed for 120 min. After filtration, the resulting nanoparticles were rinsed with purified water and backed at 200 °C for 60 min. CaF_2_ nanoparticles were immersed in an ethanol solution of ErCl_3_.6H_2_O (10 mM) at 70 °C for 60 min. After baking at 400 °C for 60 min, the core/shell structured CaF_2_/Er nanoparticles were reacted with bjSQ in chloroform solution at 25 °C for 60 min to form the interfacial complex of Er and bjSQ on the surface of CaF_2_ nanoparticles.

### 2.2. Measurements

Scanning electron microscopy (SEM) images were obtained using an Ultra-55 microscope (Carl Zeiss AG, Germany) equipped with a secondary in-lens electron detector, together with a QUANTAX detector (Bruker Corporation, Germany) for energy-dispersive X-ray spectrometry (EDS). Synchrotron X-ray powder diffraction (XRPD) patterns were obtained with a large Debye–Scherrer camera installed at the BL02B2 beamline (SPring-8), using an imaging plate as the detector and an incident X-ray wavelength of 0.9988Å. Fourier transform infrared (FTIR) spectra were measured in attenuated total reflection (ATR) mode with a diamond crystal using the Nicolet iS5 infrared spectrometer (Thermo Fisher Scientific K. K., Japan). Optical absorption and luminescence spectra were recorded on a UV-3100 spectrophotometer (Shimadzu Corporation, Japan) with an absolute specular reflectance attachment and a Jobin Yvon Fluorolog 3–22 (Horiba Scientific, Japan), respectively. A 980 nm semiconductor laser and a 671 nm diode-pumped solid-state laser with an adjustable power supplies were used as CW excitation light sources (CivilLaser, China). A 450 W Xe lamp equipped in the luminescence spectrometer (Fluorolog 3–22, Horiba Scientific, Japan) was also used as a low-intensity light source. The full width at half maximum (FWHM) at 750 nm is 350 cm^−1^. The excitation light intensity and wavelength were adjusted using neutral-density (ND) and color filters (OMG Co., Ltd., Japan). The light intensity was measured by a Si photodetector connected with a power meter (Ophir Japan Ltd., Japan).

## 3. Results and discussion

### 3.1. Formation of the interfacial complex on oxide nanoparticles

Er_2_O_3_ nanoparticles with ca. 20 nm diameter were reacted with bjSQ as an energy donor for Er^3+^ in chloroform solution, resulting in the formation of bjSQ-coordinated Er oxide nanoparticles with bright green color. The concentration of bjSQ on the oxide nanoparticle was estimated as 0.1 wt% by the absorbance change of the solution (molar concentration) after the reaction, which is sufficient to form a continuous film of dye on the nanoparticles. For comparison, core/shell structured nanoparticles with Er_2_O_3_ as a core and Yb ion as a shell layer were also prepared as before [17]. No obvious structural changes in the oxide nanoparticle were observed in SEM images (Figures 1(b) and S1) and XRPD patterns (Figure S2) upon coordination to bjSQ, i.e. it was similar to a nanoparticle with Yb^3+^ coating on the surface. This indicates that the interfacial complex composed of Er^3+^ and bjSQ is formed as a nano-ordered thin shell layer on the nanoparticle. The FTIR spectrum (Figure S3) demonstrated the formation of coordination bonds between bjSQ and Er^3+^ on the surface of the nanoparticles. The stretching vibration of the C-O bond of bjSQ around 1300 cm^–1^ shifted slightly to the lower frequency side, confirming that bjSQ coordinated with the Er^3+^ on the surface.

### 3.2. The electronic structure of dye-coordinated Ln nanoparticles

**Figure 2.**
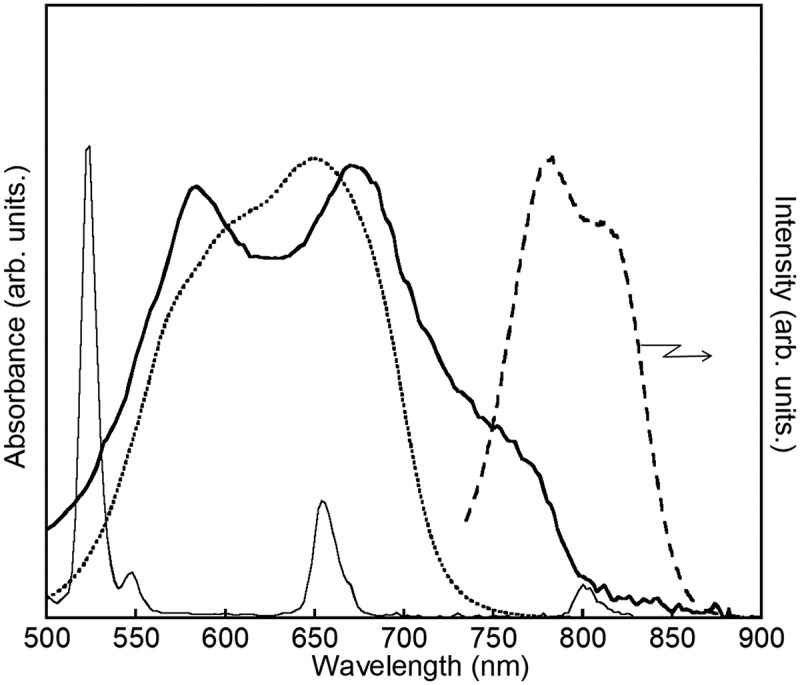
Absorption spectra of bjSQ-coordinated Er oxide nanoparticles (solid line), Er/Yb oxide nanoparticles (thin line), and bjSQ (dotted line) in the solid state. Fluorescence spectrum for bjSQ is also shown (dashed line).

The absorption spectra supported the complexation on the nanoparticle (Figure 2). The bjSQ itself shows an absorption band at around 650 nm originating from ππ* transitions. The corresponding band is observed at the lower energy side around 750 nm when coordinated to the Er ion of the nanoparticle. No luminescence band is observed in bjSQ-coordinated Er oxide nanoparticles, although the bjSQ itself shows a fluorescence band at around 800 nm. Er/Yb oxide nanoparticles show sharp absorption bands at 523.0, 547.6, 654.0, and 800.8 nm assigned to ^4^I_15/__2_→^2^H_11/2_, ^4^I_15/2_→^4^S_3/2_, ^4^I_15/2_→^4^F_9/2_, and ^4^I_15/2_→^4^I_9/2_ transitions of Er^3+^, respectively. The ^4^I_15/2_→^4^I_9/2_ transition overlaps with the fluorescence band of bjSQ around 800 nm, indicating that the energy transfer from bjSQ to the Er ion can occur. Thus, the fluorescence of bjSQ was not observed on the Er_2_O_3_ nanoparticles. At the molecular level, this interfacial complex formation may be important for the upconversion process. When a higher concentration of bjSQ (more than 5 mM) was reacted with Er_2_O_3_ nanoparticles, aggregation between particles was accelerated and the absorption and fluorescence bands of bjSQ alone appeared. This means that the interfacial energy transfer from bjSQ to Er ion, followed by the upconversion process, hardly occurs.

### 3.3. Upconversion emissions occurred at the surface of nanoparticles

**Figure 3.**
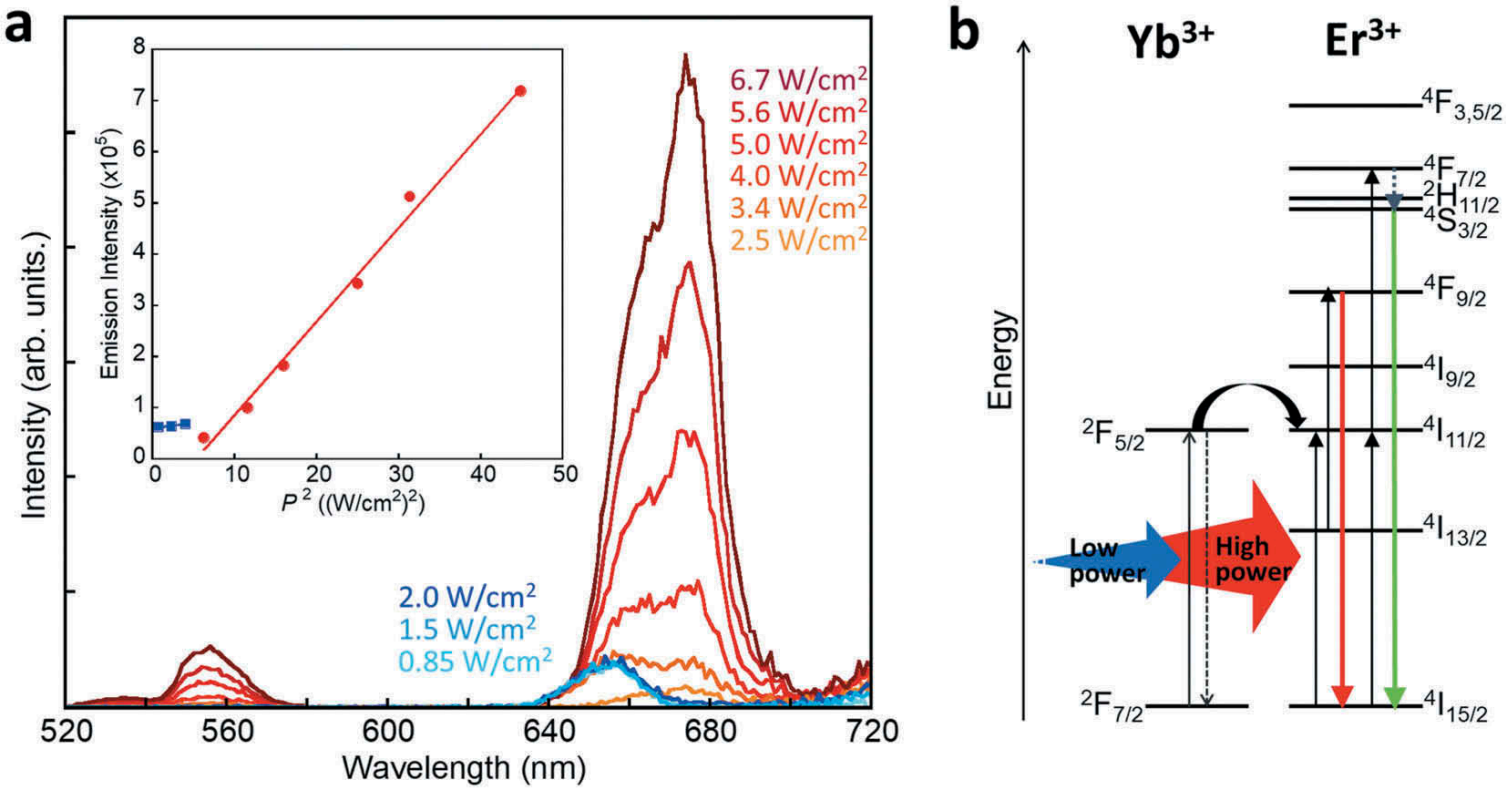
(a) Luminescence spectra of Er/Yb oxide nanoparticles in the solid state measured at different power densities using a CW laser with an excitation wavelength of 980 nm. The inset shows the relationship between the square of the excitation power density and upconversion emission intensity. (b) Schematic of the energy migration pathway.

The Er/Yb oxide nanoparticles show upconversion emissions from Er^3+^ in the core oxide by laser excitation of Yb^3+^ in the shell layer. The atomic ratio of Yb^3+^ to Er^3+^ was estimated as 0.08 by the EDS analysis. Figure 3 shows the luminescence spectra of Er/Yb oxide nanoparticles obtained through excitation by a CW laser at 980 nm. Under excitation intensities of over 2.5 W/cm^2^, the Er/Yb oxide nanoparticles exhibited luminescence bands at 555 and 674 nm assigned to the ^4^S_3/2_→^4^I_15/2_ and ^4^F_9/2_→^4^I_15/2_ transitions of Er^3+^, respectively. It is remarkable that the luminescence band position changed under intensities below 2.0 W/cm^2^ and only the red emission at 656 nm was observed. The red upconversion emission from the ^4^F_9/2_ state of Er^3+^ is induced by using two photons via the lower energy level; therefore, the upconversion emission intensity (*I*_f_) is proportional to the square of the excitation light power (*P*) [9]. In Er/Yb oxide nanoparticles, the relationship between *I*_f_ and *P* ^2^ changed between 2.0 and 2.5 W/cm^2^ as shown in the inset of Figure 3. The behavior at strong excitation intensities over 2.5 W/cm^2^ was similar to that of Er_2_O_3_ nanoparticles without Yb^3+^ shell layers (Figure S4). This suggests that the weak excitation light can only excite the Yb^3+^ as the shell layer and that excitation energy transfer may occur effectively at the surface of the Er oxide nanoparticle. However, it was difficult to observe the upconversion emission induced by the interfacial metal-to-metal energy transfer under a power excitation of less than 0.8 W/cm^2^ due to the extremely low light-absorption ability of Yb^3+^. The same results were obtained at low temperature (Figure S5), meaning that a heating effect from the laser excitation is negligible in this system.

**Figure 4.**
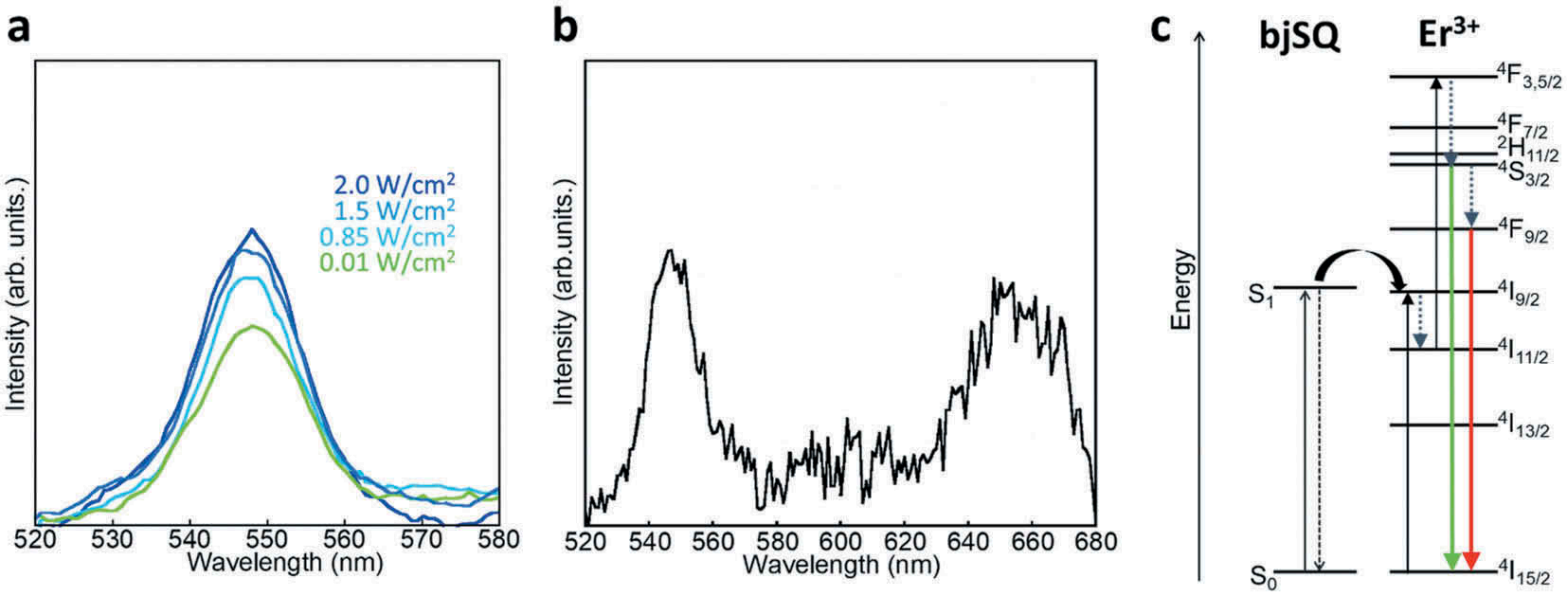
(a) Luminescence spectra of bjSQ-coordinated Er oxide nanoparticles in the solid state excited by a CW laser at 671 nm and (b) a CW Xe lamp at 750 nm. (c) Schematic of the energy migration pathway.

The bjSQ-coordinated Er oxide nanoparticles exhibit upconversion emission even under the lower power light irradiation. Figure 4(a) shows the luminescence spectra of the bjSQ-coordinated Er oxide nanoparticles under laser excitation at 671 nm. The upconversion emission originating from the ^4^S_3/2_→^4^I_15/2_ transition of Er^3+^ was observed at 548 nm even through excitation by a CW laser of 10 mW/cm^2^. The emission intensity was saturated around the excitation power of 2.0 W/cm^2^ (Figure S6), meaning that the excitation light at 671 nm was absorbed by the bjSQ existing at the nanoparticle surface. Interestingly, the green and red upconversion emissions at 548 and 650 nm were still observed by a Xe lamp with a low-power density of 1.2 mW/cm^2^ at 750 nm (Figure 4(b)). The red upconversion emission at 650 nm is assigned to the ^4^F_9/2_→^4^I_15/2_ transition of Er^3+^. Such low-power excited upconversion emissions were not observed in oxide nanoparticles without bjSQ, confirming that the upconversion on Er^3+^ occurred through the intramolecular energy transfer from bjSQ with excellent light-absorption ability at the interface. As suggested by the absorption and fluorescence spectra (Figure 2), the excitation energy on bjSQ can transfer to the upper energy level of ^4^I_9/2_ rather than that on Yb^3+^ (^4^I_11/2_), and, therefore, the green upconversion emission of Er^3+^ was enhanced rather than the red emission as shown in Figure 4(c).

### 3.4. Minimization of the energy loss with nonradiative downward relaxations in Er^3+^

The green and red upconversion emissions of Er^3+^ result from the relaxation pathways of ^4^I_9/2_→^4^I_11/2_, ^4^F_5/2_→^4^S_3/2_ (or ^2^H_11/2_), ^4^F_7/2_→^4^S_3/2_ (or ^2^H_11/2_), and ^4^S_3/2_→^4^F_9/2_, which are caused by the vibrational energy levels of the core nanoparticles. For instance, the hydroxyl groups on the surface of Ln_2_O_3_ have vibration modes around 3200–3600 cm^−1^ and 1500–1400 cm^−1^ (Figure S7) [23], which increase the probability of multiphonon relaxation. Thus, the upconversion emissions from upper energy levels such as ^2^H_9/2_, ^4^F_5/__2_, and ^4^F_7/2_ are difficult to observe on the surface of Ln_2_O_3_. To suppress such vibrational deactivation pathways, we used CaF_2_ nanoparticles as the core. CaF_2_ nanoparticles were synthesized by calcium nitrate (Ca(NO_3_)_2_) and ammonium fluoride (NH_4_F) in EDTA. CaF_2_ nanoparticles were obtained with ca. 30 nm particle size. From the FTIR spectral measurement, CaF_2_ nanoparticles had no absorption peaks in the IR region (Figure S7), which suppresses the vibrational deactivation pathway of the upconversion process. CaF_2_ nanoparticles were coated with Er^3+^ to obtain core/shell structured CaF_2_/Er nanoparticles. The atomic ratio of Er^3+^ in the shell to Ca^2+^ in the core was estimated as 0.07 by the EDS analysis. CaF_2_/Er nanoparticles were reacted with bjSQ to form the interfacial complex of Er and bjSQ on the surface of CaF_2_ nanoparticles. From the SEM image and XRPD pattern (Figure 1(c) and S8), no structural change of the CaF_2_ nanoparticles was observed by coating of Er^3+^ and coordination of bjSQ on the surface. This means that the interfacial complex composed of Er^3+^ and bjSQ is formed as a nano-ordered thin shell layer on the CaF_2_ nanoparticles, similar to that of the oxide nanoparticles.

**Figure 5.**
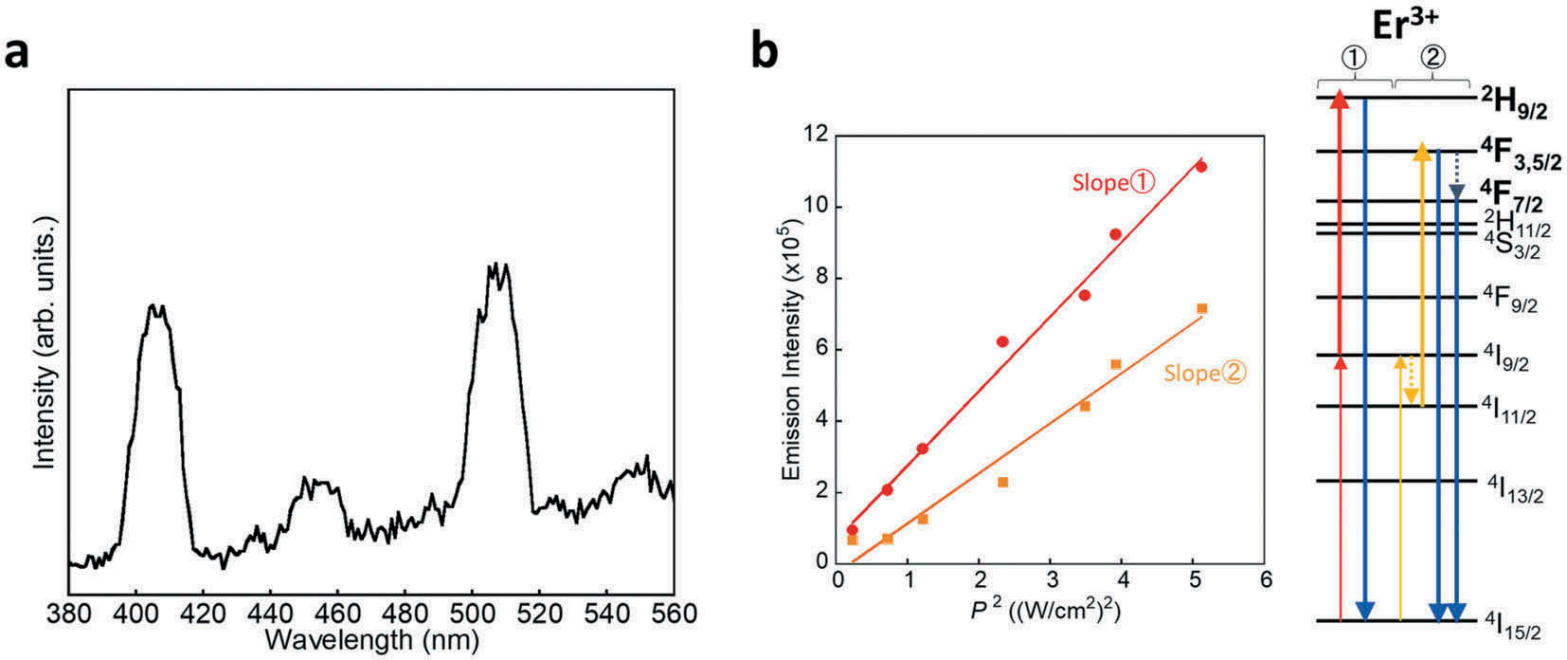
(a) Upconversion emission spectrum of bjSQ-coordinated CaF_2_/Er nanoparticles in the solid state excited by of a CW Xe lamp at 750 nm and (b) the relationship between the square of the excitation power density and upconversion emission intensity (slope 1: band line at 406 nm, slope 2: band line at 507 nm).

Figure 5(a) shows the luminescence spectrum of the bjSQ-coordinated CaF_2_/Er nanoparticles by CW Xe lamp excitation at 750 nm with a light power density of 1.2 mW/cm^2^. It is remarkable that the bjSQ-coordinated CaF_2_/Er nanoparticles successfully show upconversion emissions from the upper states at 406, 453, 507, and 549 nm assigned to ^2^H_9/2_→^4^I_15/2_, ^4^F_5/2_→^4^I_15/2_, ^4^F_7/2_→^4^I_15/__2_, and ^4^S_3/2_→^4^I_15/2_ transitions, respectively. From the plot of excitation power dependence (*P* ^2^) vs. emission light intensity (Figure 5(b)), two different upconversion pathways exist on the CaF_2_ nanoparticles: one in which the photon energy is directly upconverted to ^2^H_9/2_ from ^4^I_9/2_ after the energy transfer from bjSQ and another in which part of the excitation energy on the ^4^I_9/2_ level is deactivated to ^4^I_11/2_ followed by upconversion to the ^4^F_3/2_ or ^4^F_5/2_ level. The deactivation process from the upper energy levels of ^2^H_9/2_ and ^4^F_7/2_ to the lower energy levels of ^4^S_3/2_ and ^4^F_9/2_ was negligible since the green and red emission intensities were significantly weaker than the blue emissions. Such unusual blue upconversion emissions from the upper energy level of Er^3+^, even under low-power excitation, resulted from minimizing the energy loss by suppression of the deactivation pathway of CaF_2_.

## 4. Conclusions

In conclusion, we succeeded in controlling the multicolor upconversion luminescence of Er^3+^ by optimizing the interface in dye-coordinated nanoparticles with a core/shell structure. In bjSQ-coordinated Er oxide nanoparticles, the green and red upconversion emissions of Er^3+^ can be obtained efficiently even at a low-power light excitation of 750 nm (1.2 mW/cm^2^), in which the bjSQ works as a better light absorber than Yb^3+^. By using CaF_2_ nanoparticles as the core, nonradiative downward relaxations on the emissive energy levels of Er^3+^ can be diminished by suppression of a deactivation pathway in the core nanoparticles. As a result, unusual blue upconversion emissions from the upper energy level of Er^3+^ were successfully induced by nonlaser excitation using a CW Xe lamp at an excitation power of 1.2 mW/cm^2^. The highly efficient multicolor upconversion system investigated in this study suggests significant potential applications as future efficient photoenergy conversion systems.
