# Supplementary material for: Multicolor upconversion luminescence of dye-coordinated Er3+ at the interface of Er2O3 and CaF2 nanoparticles
Source: Sci Technol Adv Mater. 2018 Dec 18;20(1):44–50. doi: 10.1080/14686996.2018.1558911 (PMC6346724; doi:10.1080/14686996.2018.1558911)
Supplement: Supplemental Material [file TSTA_A_1558911_SM1965.pdf]

Supporting Information

Multicolor upconversion luminescence of dye-coordinated Er^{3+} at the interface of Er_2O_3 and CaF_2 nanoparticles

Ayumi Ishii, Yuya Adachi, Ayaka Hasegawa, Miyu Komaba, Shuhei Ogata, and Miki Hasegawa**

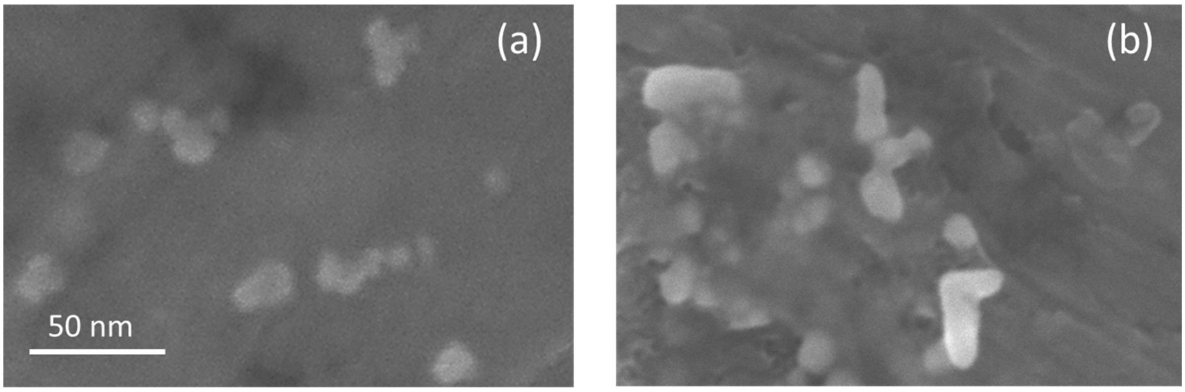

Figure S1. SEM images of (a) Er_2O_3 and (b) core/shell structured Er/Yb oxide nanoparticles.

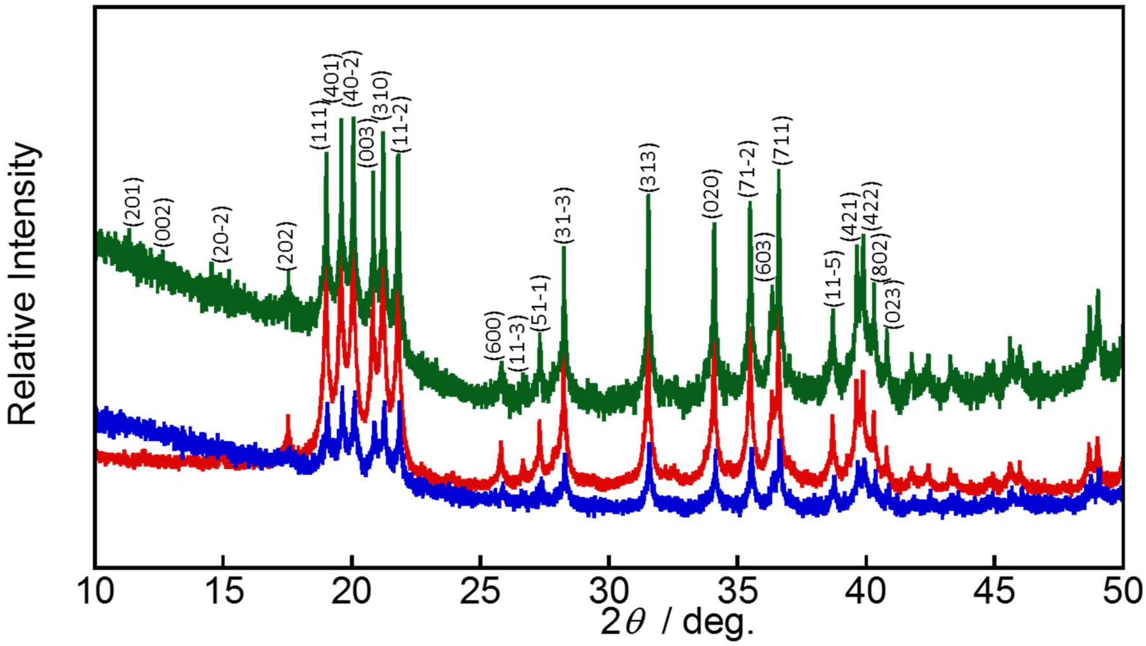

Figure S2. Synchrotron XRPD patterns (λ = 0.9988 Å) of Er_2O_3 nanoparticle (red line), Er/Yb oxide nanoparticle (green line), and bjSQ coordinated Er oxide nanoparticle (blue line).

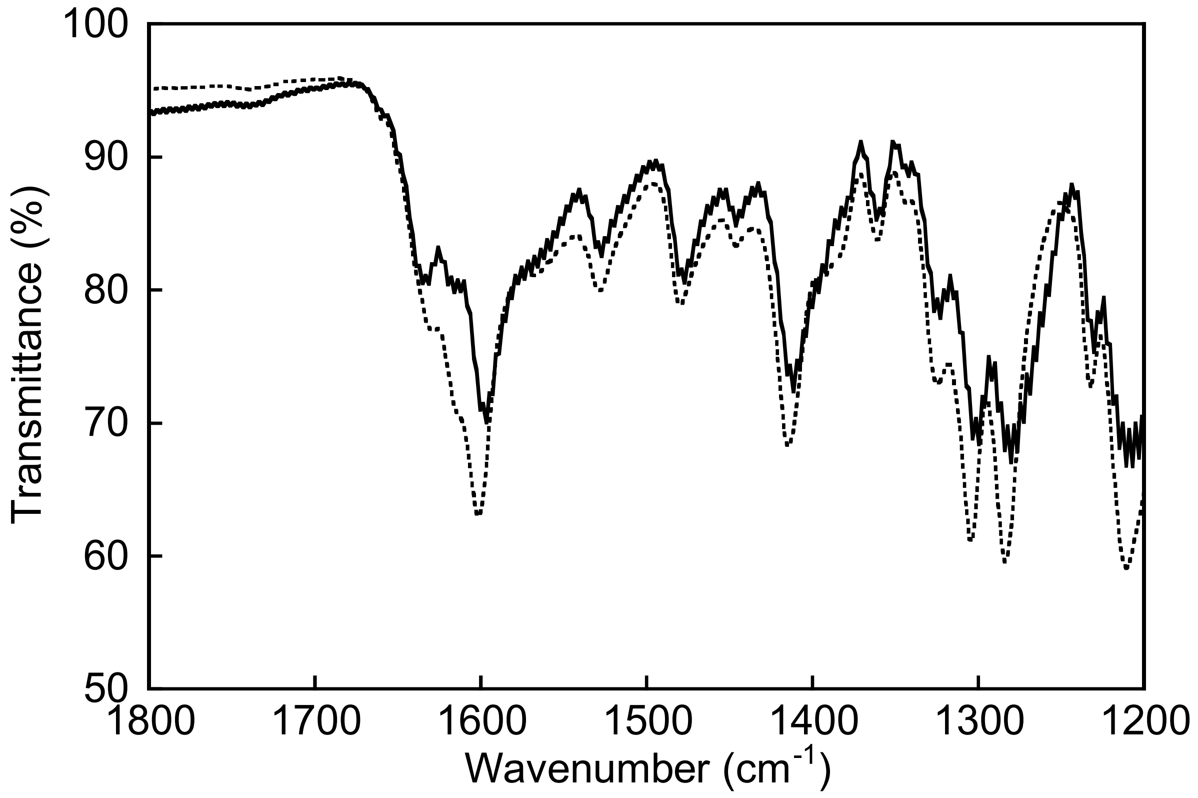

Figure S3. FTIR spectra of bjSQ itself (dotted line) and on the Er oxide nanoparticles (solid line).

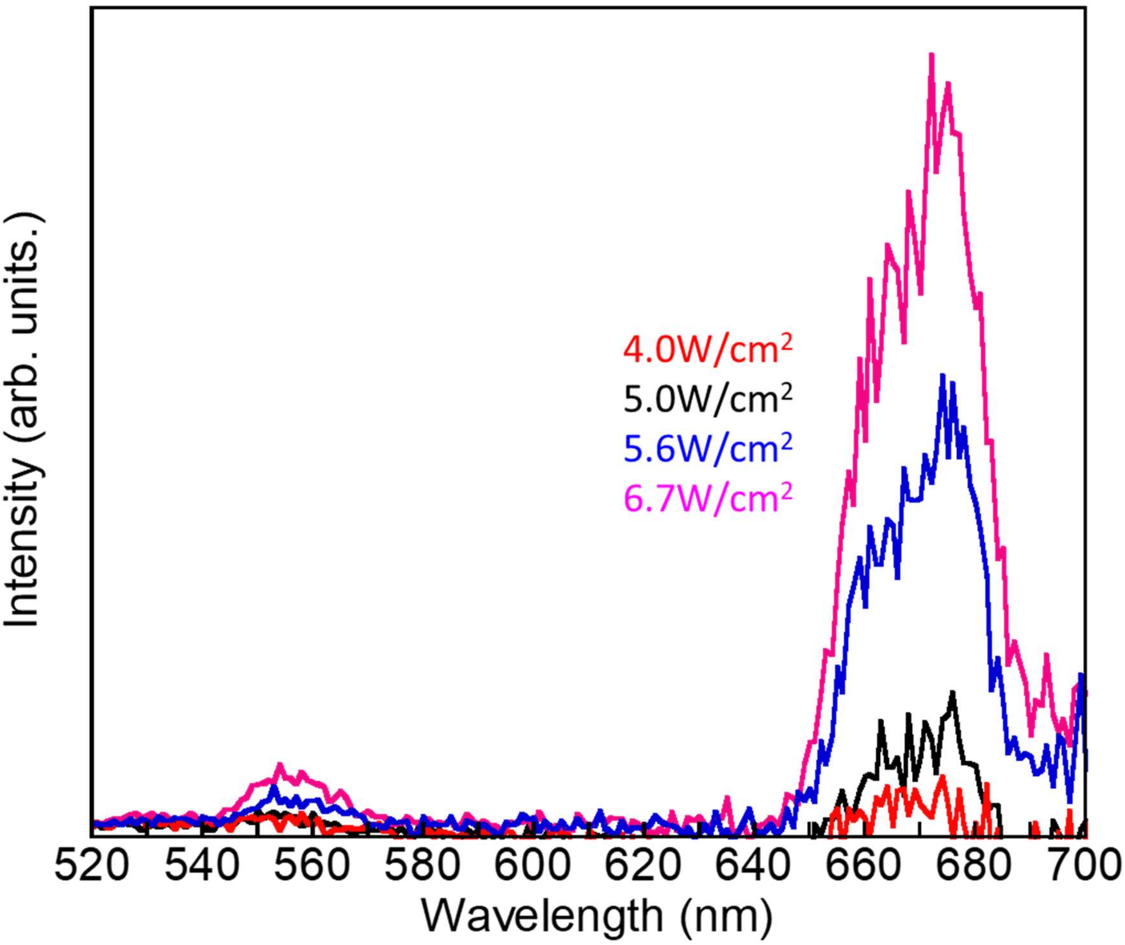

Figure S4. Up-conversion emission spectra of Er_2O_3 nanoparticles measured at different power densities using a CW laser with an excitation wavelength of 980 nm.

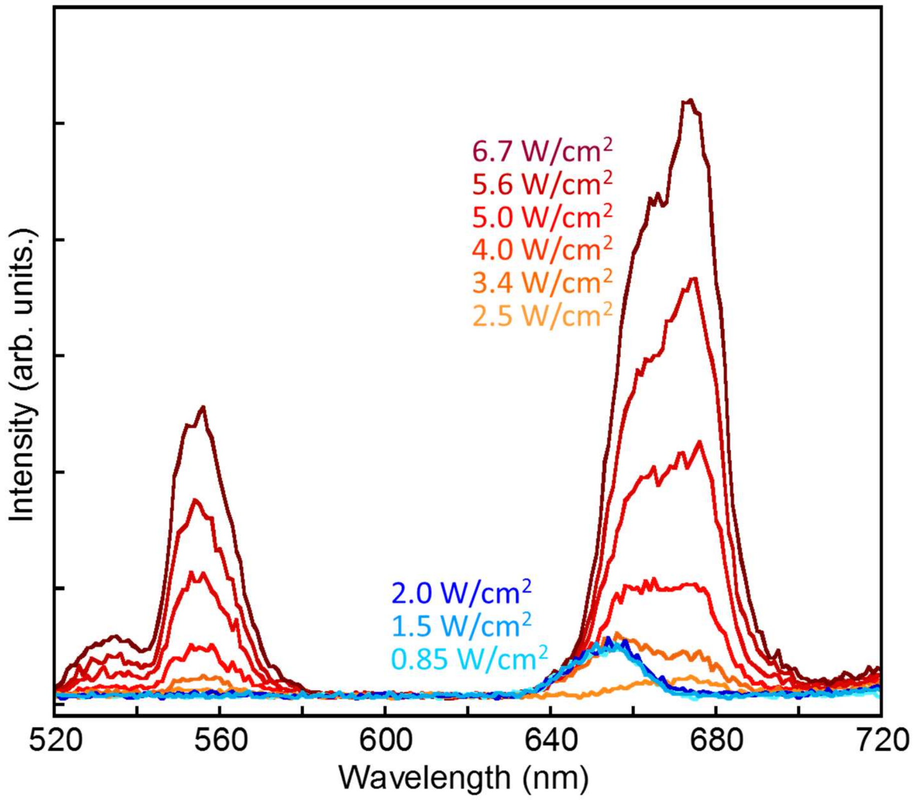

Figure S5. Up-conversion emission spectra at 77 K of Er/Yb oxide nanoparticles measured at different power densities using a CW laser with an excitation wavelength of 980 nm.

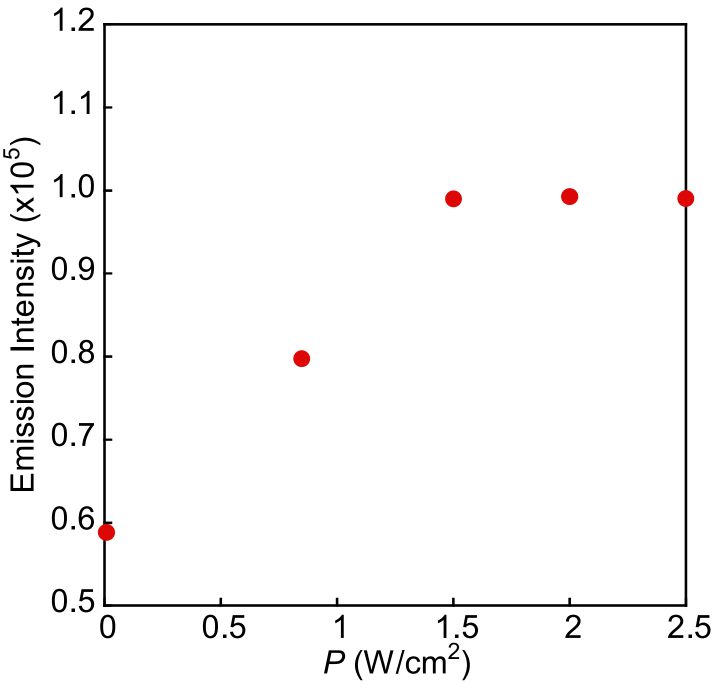

Figure S6. The relationship between the excitation power density and upconversion emission intensity of bjSQ coordinated Er oxide nanoparticles (under the laser excitation at 671 nm).

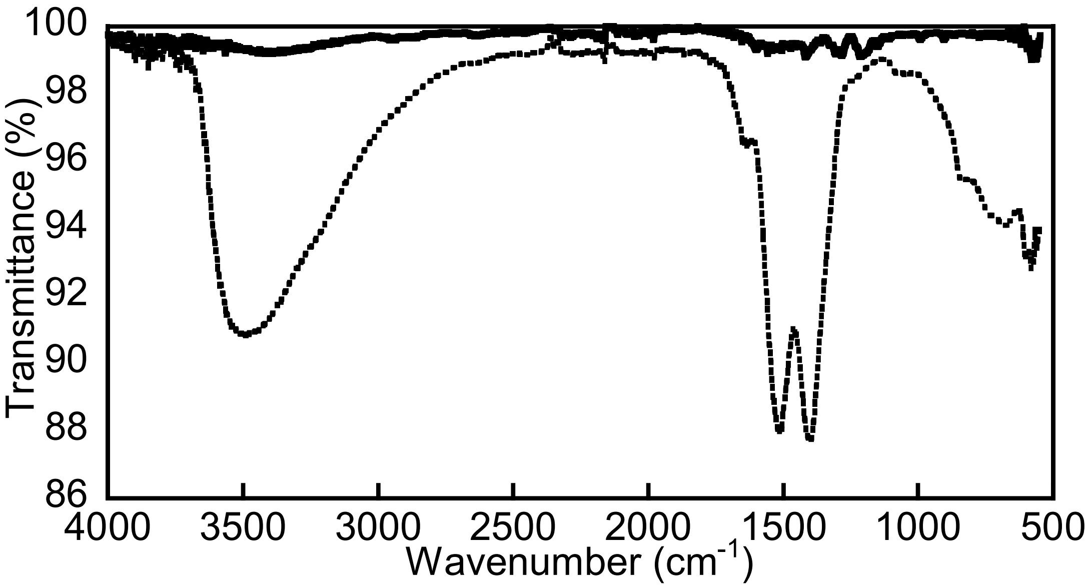

Figure S7. FTIR spectra of CaF_2 nanoparticle (solid line) and Er_2O_3 nanoparticles (dotted line).

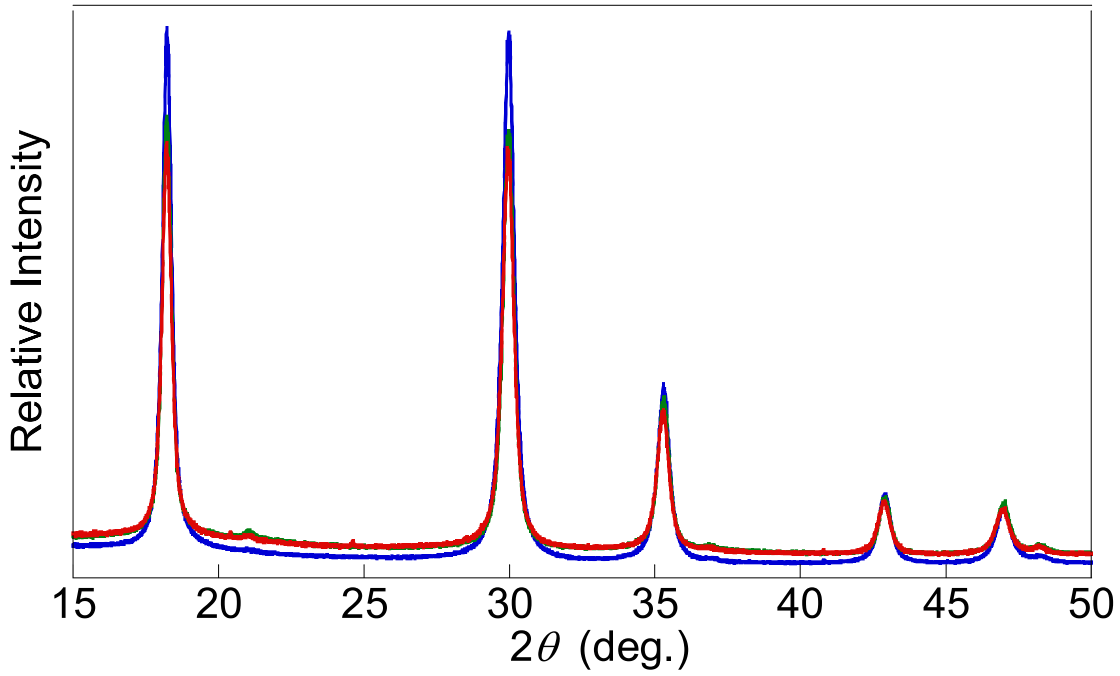

Figure S8. Synchrotron XRPD patterns (λ = 0.9988 Å) of CaF_2 nanoparticle (red line), CaF_2/Er nanoparticle (green line), and bjSQ coordinated CaF_2/Er nanoparticle (blue line).
